# One (vis‐à‐vis Planetary, Eco) health: A landscape analysis of educational programs

**DOI:** 10.1002/puh2.24

**Published:** 2022-10-17

**Authors:** Sandul Yasobant, Monal Daptardar, Karishma Krishna Kurup, Divya Panwar, Marianne Bongcac, Mara Ysabella De Los Santos, Renzo R. Guinto, Deepak Saxena, Simmi Tiwari

**Affiliations:** ^1^ Centre for One Health Education, Research, and Development (COHERD) Indian Institute of Public Health Gandhinagar (IIPHG) Gandhinagar Gujarat India; ^2^ School of Epidemiology & Public Health Datta Meghe Institute of Medical Sciences (DMIMS) Wardha Maharashtra India; ^3^ Global Health, Institute for Hygiene & Public Health (IHPH) University Hospital Bonn Bonn North Rhine‐Westphalia Germany; ^4^ Independent Veterinary Public Health Consultant Delhi India; ^5^ Chatham House London United Kingdom; ^6^ Planetary and Global Health Program St. Luke's Medical Center College of Medicine Quezon City Metro Manila Philippines; ^7^ Sunway Centre for Planetary Health Sunway University Petaling Jaya Selangor Malaysia; ^8^ National Center for Disease Control (NCDC) Ministry of Health & Family Welfare New Delhi India

**Keywords:** course, eco‐health, education, one health, planetary health

## Abstract

To sustain the health of humans, animals, and their shared environment, global advocacy in policy, funding, education, and training in multi‐actor, multi‐domain, and multi‐level collaboration is required. Emerging interdisciplinary concepts such as One Health, Planetary Health, and Eco‐Health (OH/PH/EH) have flagged opportunities to promote health across species; however, there is a lack of understanding about the extent and type of available education and training of professionals working in these disciplines. Therefore, this review aims to identify the ongoing efforts in the pedagogy of imparting OH/PH/EH education across the globe. We conducted a systematic Google database search between August and October 2021. The specific course types covered in this review were limited to degree programs and short courses. All courses using the terms “One Health,” “Planetary Health,” and “Eco health” were included. A descriptive analysis was conducted to understand the distribution of these courses based on the course type, geographic region, mode of delivery, duration, and course fees. A total of 61 courses delivered across the globe were identified in this review. Among these courses, there were three doctoral degrees, 31 master's degrees, two bachelor's degrees, and 25 short courses documented. The majority of the courses were related to OH (*n* = 43), followed by PH (*n* = 10), and EH (*n* = 08). Overall, there are still a limited number of courses or programs offered on OH/PH/EH, especially for PH and EH. There was geographic inequity as the reviewed courses were centered around the European region. There were also differences in the mode of delivery, as there was a preference for online delivery of courses, and many of them were diploma or certificate courses. Acknowledging the essence of these frameworks in tackling real‐world issues, OH/PH/EH education should be made more available in higher education to help mould OH/PH/EH‐oriented professionals.

## INTRODUCTION

Over the last two decades, there has been a growing global understanding of the relationship between human health, well‐being, animal health and the environment. This has resulted in the emergence of diverse alternative perspectives on global health research policy and practices, such as environment and health, one health (OH), planetary health (PH), biodiversity and health, eco‐social health, eco‐health (EH), and climate change and health. These emerging interdisciplinary concepts have flagged methodological opportunities for systems thinking and interdisciplinary problem‐based learning to solve these interconnected issues. However, one of the observations of these disciplines is that each field has a different perspective, and hence, their practical efforts are often off‐course, with numerous variations across the globe.

**Impact statement**

There is a need for more accessible courses in higher education to develop professionals oriented toward a transdisciplinary approach. Thus, this review highlights all the educational programs in the gambit of One Health, including Planetary and Eco health. It emphasizes the distribution of these courses by course type, geographic region, mode of delivery, duration, and course fees.

OH is a collaborative approach that recognizes nuances in the relationship between the health of humans, animals, and their shared environment to identify the socioeconomic aspects that affect this interdependence [1]. It was initially conceived as a concept [2, 3], subsequently became an approach [4, 5, 6], and has now turned into a movement [7, 8], inspiring the "One welfare" concept [9]. The ongoing coronavirus disease (COVID‐19) pandemic impels OH's evolution and its principles to prevent future epidemics [10]. OH principles have evolved to be used to prevent pandemics, for instance, against severe acute respiratory syndrome (SARS) and Middle East respiratory syndrome (MERS) [11]. The concept of OH is gaining momentum and is primarily advocated by global health agencies such as the World Health Organization (WHO) [12], Food and Agriculture Organization (FAO) [13], World Organization for Animal Health (OIE) [14], and World Bank [15] and is also being considered in the United Nations Sustainable Development Goals (SDGs) [16]. OH does not have a one‐size‐fits‐all approach, but it is a method that emphasizes sustainable collaboration between multiple disciplines to attain optimal public, animal, and environmental health and has a translational impact on global health [17]. Two other notions, PH and EH, are being used ambiguously with the concept of OH. For someone new, these concepts may easily be perceived as relatively synonymous, as all the concepts promote the underlying assumption of humans and animals sharing the same planet, and to a large extent, the same habitats, and, therefore the same environmental and health challenges [18]. The difference though subtle is in the view of health; PH focuses on human health, EH focuses on the relationship between health and ecosystems and OH considers the relationship between animal, human and environmental health [18].

Evidence indicates issues in implementing these integrated approaches because of inadequacies in policy, funding, education, training, and surveillance, suggesting the need for cross‐disciplinary knowledge, skills, and expertise, including practical experience from diverse fields [19, 20, 21]. Recent data estimate a global shortage of 12.9 million health workers, with approximately 83 countries facing major difficulties [22]. Human resource is central to implementing interventions. There has been no consensus on how to plan these interventions realistically, which would prepare students to work as One Health, Planetary Health, and Eco‐Health (OH/PH/EH) professionals locally and internationally [23].

Multiple attempts to engage students across disciplines to integrate health were made; however, there is a lack of evidence on the required skills of faculty and leadership across various health‐related fields to share knowledge and experiences to balance the collaborative perspectives in curriculum development and implementation [24]. In the last few years, various short‐term training courses have been conducted to address this gap. One such example is “the Rx One Health Summer Institute” in East Africa, which organized a training programme in 2017 which had a field‐based experiential learning course focused on OH core competency for graduate students and early career professionals from medical and veterinary schools, agriculture, environmental sciences, and conservation professionals [25]. Such interdisciplinary courses bridge professionals from various disciplines and create an understanding of inter‐ and transdisciplinary collaborative approaches. However, isolated evaluations of the growing pool of OH/PH/EH academic programs have identified gaps in the competencies included in these programs. Emphasis has been placed on infectious disease threats and has failed to recognize the broader application of these concepts. The current review aims to identify ongoing global efforts in the pedagogy of imparting OH, PH, and EH education.

## METHODS

### Search strategy

We conducted a systematic Google database search from August to October 2021 using the different key terms “One Health,” “Planetary Health,” and “Eco‐Health.” For the second construct, various combinations of the following terms about educational program types were used: “courses,” “diploma,” “degree,” “bachelor,” “master,” “graduate,” “post‐graduate,” “doctoral degree,” “short courses,” “e‐Course,” “distance courses,” and “online course.” In addition, the web pages of universities, academic institutes, donor agencies, multilateral agencies, and commissions working in OH/PH/EH were identified and accessed for further extraction of courses that could have been missed in the first Google search.

### Inclusion and exclusion criteria

This review covers specific academic programs and is limited to degree programs and short courses. By "courses" we mean all the educational programs having the terms “One Health,” “Planetary Health,” and “Eco health” were included in the review. Only courses described in the English language were included.

We excluded courses on “One Medicine” as we intended to identify the broader aspects of health, OH/PH/EH modules within the human or veterinary medicine curriculum. Webinars/conferences/pre‐conference workshops conducted on OH/PH/EH were excluded, as were new courses that were scheduled to be launched beyond the search period and courses that may resemble OH/PH/EH but did not consist of the terms “One Health” or “Planetary Health” or “Eco‐Health.”

### Study definitions

#### One health

The One Health definition developed by the OHHLEP states:

“One Health is an integrated, unifying approach that aims to sustainably balance and optimize the health of people”, animals and ecosystems. It recognizes the health of humans, domestic and wild animals, plants, and the wider environment (including ecosystems) are closely linked and inter‐dependent. The approach mobilizes multiple sectors, disciplines and communities at varying levels of society to work together to foster well‐being and tackle threats to health and ecosystems, while addressing the collective need for clean water, energy and air, safe and nutritious food, taking action on climate change, and contributing to sustainable development’ [26]

The One Health Commission defines One Health as:

“…the collaborative effort of multiple health science professions, together with their related disciplines and institutions—working locally, nationally, and globally—to attain optimal health for people, domestic animals, wildlife, plants, and our environment.”[27]

The One Health Global Network defines One Health as an approach:

“…to improve health and wellbeing through the prevention of risks and the mitigation of effects of crises that originate at the interface between humans, animals, and their various environments.” [28]

#### Planetary health

PH is defined by the Lancet‐Rockefeller Commission as:

“…the achievement of the highest attainable standard of health, wellbeing, and equity worldwide through judicious attention to the human systems—political, economic, and social—that shape the future of humanity and the Earth's natural systems that define the safe environmental limits within which humanity can flourish. Put simply, planetary health is the health of human civilization and the state of the natural systems on which it depends.” [29]

#### Eco health

EcoHealth is defined by the EcoHealth Alliance as a field that:

“…is committed to fostering the health of humans, animals, and ecosystems and to conducting research that recognizes the inextricable linkages between the health of all species and their environments. A basic tenet held is that health and wellbeing cannot be sustained in a resource depleted, polluted, and socially unstable planet” [30]

#### Short courses

Are coursework such as certificates or diploma programs offered by an academic institute or professional bodies/alliances/associations with or without any credits.

#### Degree courses

They are undergraduate, postgraduate, master's, or doctoral degrees provided by higher educational institutes.

### Data extractions and analysis

Two sets of reviewers (S.Y. and D.P.) and (M.B. and M.Y.S.) extracted the data from eligible studies to summarize and tabulate the essential findings. Two other reviewers (DS and RG) validated the search results. Finally, three reviewers (S.Y., K.K.K., and M.D.) evaluated the scope of each course. The information was added to an Excel spreadsheet to synthesize the data. The data were extracted as the type of course, course length, mode of course delivery, course start date, geography of the institute offering the course, and course fees in US dollars (USD). The information was further analysed to describe the courses by year, duration of the program, geographical location of the WHO region, course delivery, and cost of enrolling in these courses.

## RESULTS

### General description

A total of 61 courses delivered across the globe were identified in this review. The majority of the courses were related to OH (*n* = 43), followed by PH (*n* = 10), and EH (*n* = 08). Of these courses, there were three doctoral degrees, 31 master's degrees, two bachelor's degrees, and 25 short courses documented.

Considering the type of course, approximately 34.9% of OH courses and 37.5% of EH courses are offered as diplomas/certificates, whereas 30% of PH courses are short online courses. There are only two bachelor's courses, one on OH and another on PH. In masters, about 66.6% were on OH, whereas 22.2% were on PH and only 11.1% were on EH. In terms of a doctoral degree, there are only three courses available, all of which are from the OH domain.

**FIGURE 1:**
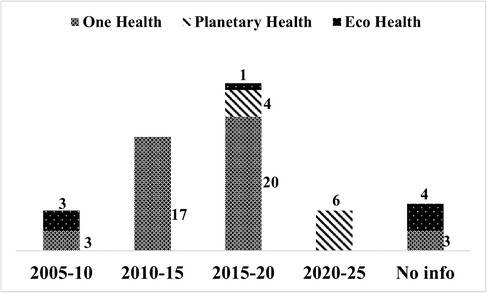
The year of onset of the OH/PH/EH educational courses, irrespective of their type. Footnote: Seven courses have no information regarding the year of launch

The first course on OH and EH was launched in 2008, whereas the first course on PH was launched in 2017 (Figure 1). This indicates the emergence of concepts and the need for new courses. In 2021, there was an increase in the number of PH courses, with six courses launched in that year, indicating a growing demand. However, we identified seven courses without any information regarding the year of launch.

#### Geographic distributions

**FIGURE 2:**
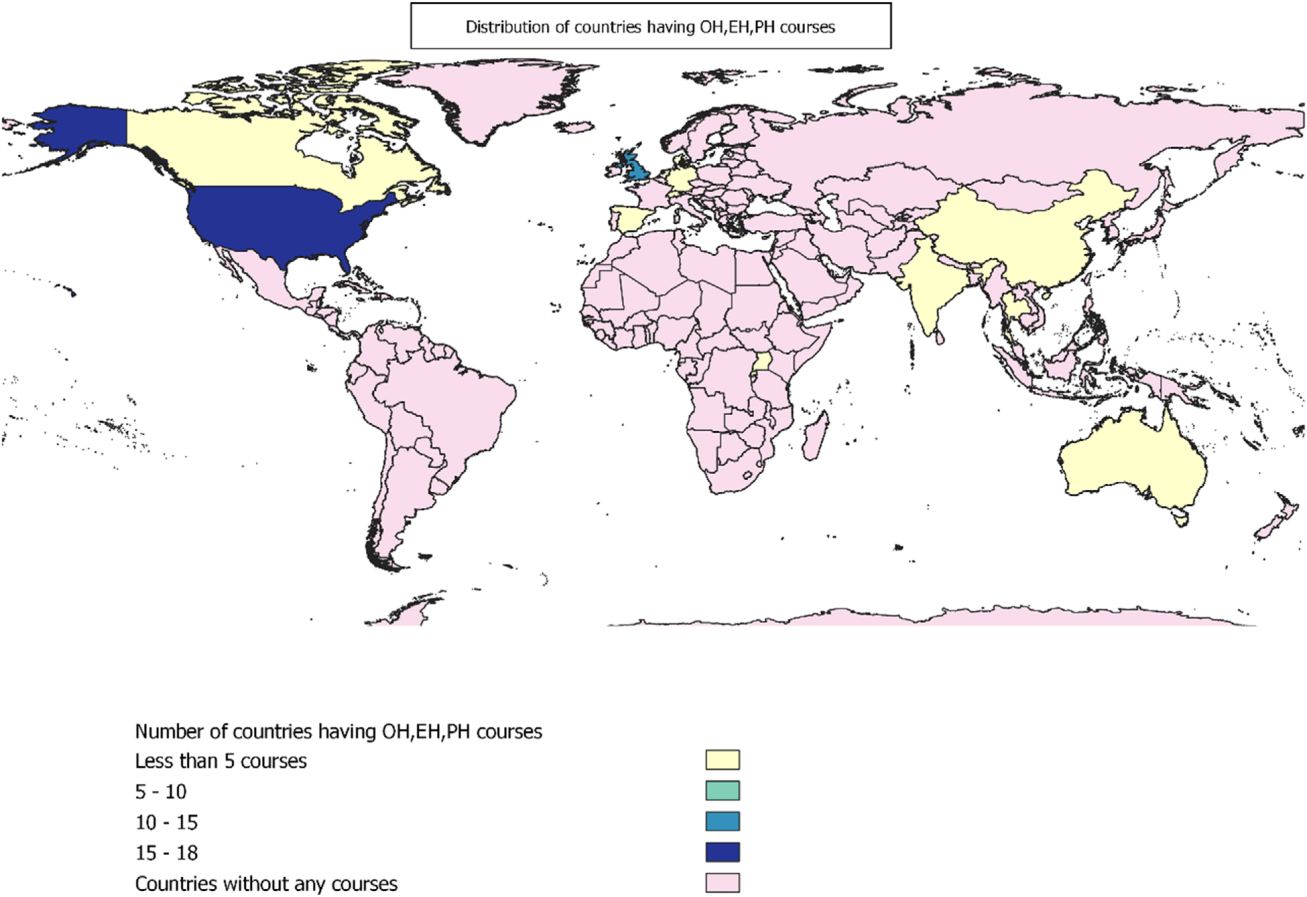
Distribution of OH, PH, EH courses as per the country, Software‐Open source software, QGIS, Source of shape file‐https://international.ipums.org/international/gis.shtml

With regard to the geographic distribution of the courses (Figure 2), most (39%) were delivered from Europe, followed by 38% from North America, 10% from Asia, 7% from Oceania, 3% from Africa, and 2% from the Caribbean. The OH/PH/EH courses were geographically distinct across their distribution. Most of the OH courses are offered in North America (46.5%), whereas most of the PH courses (70%) and EH courses (50%) are offered in Europe. Approximately 30.2% of OH courses are offered in the European region. Approximately 40% of the short courses are offered from North America, whereas 40.9% of diploma courses and 57.1% of courses are from the European region.

#### Course delivery and duration

**FIGURE 3:**
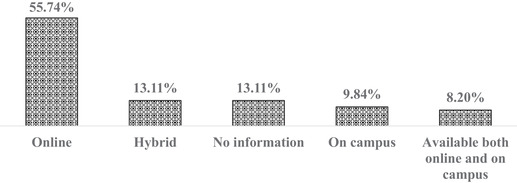
Mode of OH/PH/EH course delivery

Four different modes of course delivery were documented (Figure 3). The course delivery was either online, on‐campus, hybrid mode, or both on‐campus and online. The majority (56%) of courses were delivered through an online platform, with OH (51.2%), PH (60%), and EH (75%) courses. Only 10% of courses are delivered on campus, 13% through the hybrid mode, and 8% of courses are available both online and on‐campus. Thirteen% of these courses, regardless of the domains (OH, EH, and PH), had no information on course delivery available on the website. Most of the short courses (68%) and diploma courses (63.6%) were offered online.

**FIGURE 4:**
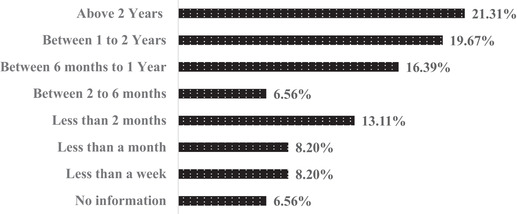
OH/PH/EH courses by their duration

The average duration (Figure 4) of short courses was 12 weeks, ranging from <1 to 104 weeks. About 4% of the short courses, have 1–2 years of time duration, 8% had 6 months to 1 year of course duration, 8% had 2–6 months of course duration, 32% had 1–2 months of course duration, 16% had 1 week to 1 month of course duration, 20% had 1 week or less than 1 week of course duration, and 12% of short courses had no information about the duration. About 54.5% of diploma courses are offered, with an average length of more than 1 year. About 63.6% of diploma courses are offered online.

#### Course fees

**FIGURE 5:**
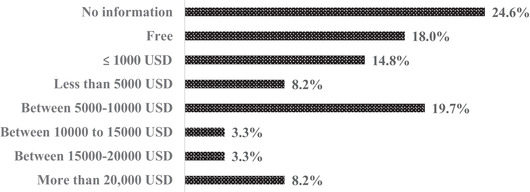
OH/PH/EH course fees (standardized to USD)

The average course fee was 10,743 USD ranging between 14 USD to 56,724 USD (Figure 5). Eleven courses had fees more than the average course fees. These course fees also varied between the type of students; for example, there were different fees for local and international students. We also found that 18% of the courses were free of charge. However, 24% had no information on fees. Evaluating by type of domain, in the OH domain, 18.6% of courses were offered as free, while 20% from PH and 12.5% from EH were offered free of cost. We found that 40% of short courses were free of cost and 24% had a course fee of ≤1000 USD. As the course duration increased, there was a proportionate increase in fees. Close to 30% of the degree courses have a course fee of >20,000 USD.

Table 1 summarizes OH/PH/EH courses and their characteristics in the form of geographical distribution, type of course, mode of delivery, and course fees. Table 2 indicates the same characteristics by course type.

**TABLE 1:** Analysis of course by health domains, 2021

	**OH**	**PH**	**EH**
**Characteristics**	***n* = 43 (%)**	***n* = 10 (%)**	***n* = 8 (%)**
**Geographic distribution**			
Africa	2 (4.7)	–	–
Asia	5 (11.6)	1 (10)	–
Caribbean	1 (2.3)	–	–
Europe	13 (30.2)	7 (70)	4 (50)
North America	20 (46.5)	1 (10)	2 (25)
Oceania	2 (4.7)	1 (10)	1 (12.5)
No information	–	–	1 (12.5)
**Type of course**			
Short course‐in person	3 (7)	–	1 (12.5)
Short course‐online	12 (27.9)	3 (30)	2 (25)
Short course‐hybrid	3 (7)	–	1 (12.5)
Diploma/Certificate	15 (34.9)	4 (4)	3 (37.5)
Bachelor	1 (2.3)	1 (10)	–
Master	6 (14)	2 (20)	1 (12.5)
Doctorate	3 (7)	–	–
**Mode of delivery**			
Available on both online and on campus	5 (11.6)	–	–
Hybrid	4 (9.3)	3 (30)	1 (12.5)
On campus	5 (11.6)	1 (10)	–
Online	22 (51.2)	6 (60)	6 (75)
No information	7 (16.3)	–	1 (12.5)
**Fees of course (standardized USD)**			
Free	8 (18.6)	2 (20)	1 (12.5)
≤1000	6 (14)	1 (10)	2 (25)
1001–5000	4 (9.3)	1 (10)	–
5001–10000	7 (16.3)	2 (20)	3 (37.5)
10001–15000	1 (2.3)	1 (10)	–
15001–20000	2 (4.7)	–	–
>20000	5 (11.6)	–	–
No information	10 (23.3)	3 (30)	2 (25)

**TABLE 2:** Descriptive analysis of reviewed courses by type of course, that is, short, diploma, bachelor degree or higher courses, 2021

	**Short courses**	**Diploma courses**	**Degree courses**
**Characteristics**	***n* = 25 (%)**	***n* = 22 (%)**	***n* = 14 (%)**
**Domains**			
One Health	18 (72)	15 (68.2)	10 (71.4)
Planetary Health	3 (12)	4 (18.2)	3 (21.4)
**Geographic distribution**	4 (16)	3 (13.6)	1 (7.1)
Africa	1 (4)	–	1 (7.1)
Asia	3 (12)	3 (13.6)	–
Caribbean	1 (4)	–	–
Europe	7 (28)	9 (40.9)	8 (57.1)
North America	10 (40)	8 (36.4)	5 (35.7)
Oceania	2 (8)	2 (9.1)	–
No information	1 (4)	–	–
**Mode of delivery**			
Available on both online and on campus	2 (8)	1 (4.5)	2 (14.3)
Hybrid	3 (12)	2 (9.1)	3 (21.4)
On campus	2 (8)	1 (4.5)	3 (21.4)
Online	17 (68)	14 (63.6)	3 (21.4)
No information	1 (4)	4 (18.2)	3 (21.4)
**Fees of Course (standardized USD)**			
Free	10 (40)	–	1 (7.1)
≤1000	6 (24)	3 (13.6)	–
1001–5000	1 (4)	2 (9.1)	2 (14.3)
5001–10000	1 (4)	9 (40.9)	2 (14.3)
10001–15000	–	1 (4.5)	1 (7.1)
15001–20000	–	1 (4.5)	1 (7.1)
>20000	–	1 (4.5)	4 (28.6)
No information	7 (28)	5 (22.7)	3 (21.4)

## DISCUSSION

The concepts of OH/PH/EH have been used interchangeably to foster recurrent global discussions regarding their importance. Our current review is unique in that it evaluates all the courses available under these health domains to identify their geographical presence, accessibility, and availability to the health workforce. Overall, there are still a limited number of courses or programs offered on OH/PH/EH, especially for PH and EH. However, more offerings might be available, but are not easily accessible or searchable online, unlike the courses reviewed in this study. During the data extraction, it was observed that some educational and institutional websites were outdated and incomplete. Additionally, some educational institutions may not have established online platforms. With this said, we find that most of the health domain courses are centred around the European region. There seems to be a preference for the online delivery of courses, and a large number of them are diploma or certificate courses. It would be encouraging to identify the relatively free courses available in OH domain. However, our review highlights the opportunity for more training in planetary and EH domains.

At the outset, there seems to be strong support for developing and executing inter‐collaborative health approaches [31]. However, there is still a lack of global initiatives to develop and ensure sustainable knowledge in the OH, PH, and EH domains. The reviewed courses recognize the need for interdisciplinary collaboration, but many programs are still limited to health‐related disciplines or professions. They are either taught and available to medical or veterinary/animal health professionals and offer a track for further specialization. One positive characteristic is that attempts have been made to holistically incorporate social, cultural, economic, and environmental aspects even in these seemingly specialist‐leaning courses. More effort is needed to ensure that other fields and disciplines outside of human health, animal health, and environmental sciences, such as the social sciences, humanities, and indigenous perspectives, are genuinely incorporated into the courses.

Through this review, we identified a stark disparity in the geographic distribution of health‐domain courses. The European and North American regions led OH, PH, and EH courses compared to Asia and Africa, illustrated how awareness and expertise on OH/PH/EH are highly concentrated and isolated in high‐income country settings. This disproportionate distribution of knowledge platforms is concerning. Interestingly, there is a dearth of such programs in regions of the Global South, such as Africa, Asia, and Latin America, which are notable for their high biodiversity and greater vulnerability to zoonotic spillovers and climate change [32]. While the availability of such training programs intends to trickle techniques and expertise to the Global South, however, this strategy fails in the absence of an enabling environment to utilise the new skills [33].

Almost 30% of degree courses charged a fee of more than 20,000 USD and the average course fee is more than 10,000 USD, hence posing a challenge of affordability for global south candidates. The world has witnessed multiple diseases transcending geopolitical boundaries; however, our review identifies the inequality in the training platforms of such courses, especially for professionals from developing nations. Ensuring the availability and accessibility of these courses in developing nations would be a key future of the OH/PH/EH courses. Establishing more courses in biologically diverse areas would enable technical capacity development and potentially catalyse the discovery of new knowledge. While there is increasing coordination for OH projects, there clearly exists a dearth of research capacity‐building programs in OH that were previously evidenced in South Asia; however, there still seems to be a lack of investments in this direction [34]. The review identified most of the courses in the health domains to be delivered online. The use of digital technologies in the delivery of these courses enables multidisciplinary and cross‐boundary access to knowledge. OH course that allows interaction among international students was identified to reduce cultural biases and provide an environment for open learning and enhanced collaboration [35]. Although an online approach has been adopted with the aim of accessibility, e‐learning strategies depend on students’ affinity with technology [35]. The lack of face‐to‐face in‐class interactions, inability to implement complex concepts, and learning from practice could hinder the learning process of enrolled students [35]. Internet connectivity in low‐income countries must also be enhanced so that students from these settings can benefit from the digitalization of OH/PH/EH education.

While this review focused on formal OH/PH/EH education offered by academic institutions, usually intending to develop professionals and experts, it is crucial to recognize that extensive education and advocacy for OH/PH/EH needs to be undertaken in communities. Although establishing more OH/PH/EH courses would produce more professionals who can craft policies, conduct research, or invent solutions, building OH/PH/EH literacy among the public will ensure that policies and solutions have the backing of community support and are effectively implemented in the real world. Such heightened literacy will also help combat "infodemics" or epidemics of misinformation, especially on online platforms, which have complicated the global response to the COVID‐19 pandemic [35]. This pandemic also boosted capacity‐building projects as part of the collaborative OH educational programs (i.e., PANDORA and AFROHUN). Another area of enhancement that was not prominently observed in the reviewed OH/PH/EH programs from our study is the collaboration between Global North and Global South institutions and students. Collaboration between academic institutions in the global north and field setting in the global south are successful models that could be replicated and bidirectional arrangements would enrich OH/PH/EH learning, especially those places at the frontlines of OH/PH/EH challenges [36]. These innovations will decentralize OH/PH/EH education from the Global North and truly "decolonize" health education in general, which is now a growing discourse in the global health community [37].

This review provides a comprehensive resource of the training programs in OH, EH, and PH in the global domain and an opportunity for stakeholders and professionals to develop and strengthen future training programs. Our study also highlights the disparity in courses for OH between the global south and the global north and the need for capacity‐building partnerships. However, our study has a few limitations. First, we reviewed only the descriptions of courses that are accessible on online platforms. Nonetheless, a comprehensive and validated search was conducted to ensure the completeness and accuracy of all the collected information. Second, there might have been courses that were not listed on online databases, which would may have been missed in the review.

COVID‐19 has registered the need to protect biodiversity to protect human health. This must create more opportunities for educational institutions as the world adapts to a better understanding of ecosystem. Our review identified most of the courses were for OH [38] domain specifically. Therefore, institutions have an opportunity to develop courses emphasizing on EH/PH, with particular emphasis on the unifying OH concept, which remains a key to global sustainability [38]. There has been an increase in capacity development program globally for increasing pandemic preparedness and Resilience through programs such as Epidemic Intelligence service programs—EIS and Field Epidemiology Training Program—FETPs traditionally meant for in‐service medical and Veterinary professionals [39]. Maybe expanding the portfolio of such courses and including students from professional disciplines could prepare the future generations of frontline professionals competent in OH/EH/PH.

## CONCLUSION

In this review, we report a disproportionate growth of educational opportunities, both short courses, and professional degrees in OH/PH/EH, specifically centred in developed countries. The availability of online programs improves accessibility; however, there are concerns about the implementation and practice of these competencies by trained professionals. Acknowledging that the essence of this training is tackling real‐world problems, the OH/PH/EH education should be accessible at the undergraduate level, to develop professionals oriented in a transdisciplinary OH/PH/EH approach at a much earlier stage. Furthermore, the training framework must become accessible to a wide range of disciplines and professions rather than monopolized by the health sciences (e.g., medicine, public health, and veterinary medicine). We recommend developing training for all health domains, centred in developing nations.

## AUTHOR CONTRIBUTIONS

Conceptualization: Sandul Yasobant, Monal Daptardar, Karishma Krishna Kurup, Divya Panwar, Marianne Bongcac, Mara Ysabella De Los Santos, Renzo R. Guinto, Deepak Saxena, Simmi Tiwari; Data extraction: Sandul Yasobant, Monal Daptardar, Karishma Krishna Kurup, Divya Panwar, Marianne Bongcac, Mara Ysabella De Los Santos, Renzo R. Guinto; Formal analysis: Sandul Yasobant, Monal Daptardar, Karishma Krishna Kurup, Divya Panwar, Marianne Bongcac, Mara Ysabella De Los Santos, Renzo R. Guinto; Writing‐original draft: Sandul Yasobant, Monal Daptardar, Karishma Krishna Kurup, Divya Panwar, Marianne Bongcac, Mara Ysabella De Los Santos, Renzo R. Guinto, Deepak Saxena, Simmi Tiwari; Writing‐review & editing: Sandul Yasobant, Monal Daptardar, Karishma Krishna Kurup, Divya Panwar, Marianne Bongcac, Mara Ysabella De Los Santos, Renzo R. Guinto, Deepak Saxena, Simmi Tiwari. All authors approved the final draft.

## CONFLICTS OF INTEREST

The authors report no conflicts of interest in this work.

## ETHICS STATEMENT

Ethics approval for this study was not required, as this study did not involve any human or animal participants. This is secondary to its nature.

## Data Availability

All relevant data supporting this study's findings are within the manuscript.
